# Overexpression of ABCB1 Associated With the Resistance to the KRAS-G12C Specific Inhibitor ARS-1620 in Cancer Cells

**DOI:** 10.3389/fphar.2022.843829

**Published:** 2022-02-23

**Authors:** Xing-Duo Dong, Meng Zhang, Chao-Yun Cai, Qiu-Xu Teng, Jing-Quan Wang, Yi-Ge Fu, Qingbin Cui, Ketankumar Patel, Dong-Tao Wang, Zhe-Sheng Chen

**Affiliations:** ^1^ Department of Pharmaceutical Sciences, College of Pharmacy and Health Sciences, St. John’s University, Queens, NY, United States; ^2^ Department of Traditional Chinese Medicine, Shenzhen Hospital, Southern Medical University, Shenzhen, China; ^3^ School of Public Health, Guangzhou Medical University, Guangzhou, China; ^4^ Department of the Ministry of Science and Technology, Guangxi International Zhuang Medicine Hospital, Nanning, China

**Keywords:** ARS-1620, KRAS-G12C inhibitor, multidrug resistance, ABC transporter, ABCB1

## Abstract

The KRAS-G12C inhibitor ARS-1620, is a novel specific covalent inhibitor of KRAS-G12C, possessing a strong targeting inhibitory effect on KRAS-G12C mutant tumors. Overexpression of ATP-binding cassette super-family B member 1 (ABCB1/P-gp) is one of the pivotal factors contributing to multidrug resistance (MDR), and its association with KRAS mutations has been extensively studied. However, the investigations about the connection between the inhibitors of mutant KRAS and the level of ABC transporters are still missing. In this study, we investigated the potential drug resistance mechanism of ARS-1620 associated with ABCB1. The desensitization effect of ARS-1620 was remarkably intensified in both drug-induced ABCB1-overexpressing cancer cells and ABCB1-transfected cells as confirmed by cell viability assay results. This desensitization of ARS-1620 could be completely reversed when co-treated with an ABCB1 reversal agent. In mechanism-based studies, [^3^H] -paclitaxel accumulation assay revealed that ARS-1620 could be competitively pumped out by ABCB1. Additionally, it was found that ARS-1620 remarkably stimulated ATPase activity of ABCB1, and the HPLC drug accumulation assay displayed that ARS-1620 was actively transported out of ABCB1-overexpressing cancer cells. ARS-1620 acquired a high docking score in computer molecular docking analysis, implying ARS-1620 could intensely interact with ABCB1 transporters. Taken all together, these data indicated that ARS-1620 is a substrate for ABCB1, and the potential influence of ARS-1620-related cancer therapy on ABCB1-overexpressing cancer cells should be considered in future clinical applications.

## Introduction

Multidrug resistance (MDR) has long been considered a major barrier to the success of cancer chemotherapy since it enhanced the survival of cancer cells by attenuating the effectiveness of anticancer drugs (Shen et al., 2008; Paškevičiūtė and Petrikaitė, 2019). The ATP-binding cassette (ABC) transporters, which consist of seven classes of membrane proteins, are strongly associated with MDR, as the overexpression of ABC transporters is deemed to be the dominant contributor to MDR. ABC transporters act as efflux pumps on the cell membrane and their primary function is to protect diverse organs such as the intestine or kidney by pumping out toxins and xenobiotics (Dassa and Bouige, 2001; Al-Ali et al., 2019). The overexpression of ABC transporters in cancer cells accelerates the efflux of chemotherapeutic drugs, resulting in cancer drug resistance, recurrence and ultimately death in cancer patients (Gillet and Gottesman, 2010; Wu et al., 2011). Several predominant and most well-characterized ABC transporters, such as ABCB1 (P-glycoprotein, P-gp; multidrug resistance 1, MDR1), ABCG2 (breast cancer resistance protein, BCRP; mitoxantrone resistance, MXR), and ABCC1 (multidrug resistance protein 1, MRP1) have been extensively studied on MDR (Dean, 2009; Li et al., 2016; Robey et al., 2018). ABCB1 was the first recognized and examined ABC transporter (Juliano and Ling, 1976). The wide use of chemotherapeutic drugs that are substrates of ABCB1 including paclitaxel and doxorubicin could lead to the activation/overexpression of ABCB1, at the same time, the overexpression of ABCB1 confers resistance to these drugs in cancer cells and cancer patients (Genovese et al., 2017). What more serious is that this acquired drug resistance may shift to other kinds of substrates of ABCB1 and a growing number of molecularly targeted chemotherapeutic drugs have been identified as substrates for ABCB1 at present, for example, mTOR inhibitor WYE-354 (Wang J. et al., 2020), histone deacetylase six inhibitor ricolinostat (Wu et al., 2018), or ALK tyrosine kinase inhibitor ceritinib (Katayama et al., 2016). In addition, ABCB1 is broadly present in the gastrointestinal tract, the blood-brain barrier, liver, and kidney, and the overexpression of it in these sites certainly has considerable clinical significance for the application of all ABCB1 substrates (Gottesman et al., 2002; Szakács et al., 2006). Therefore, determining whether existing anticancer drugs are ABCB1 substrates could predict the treatment outcome to develop improved cancer therapy regimens *via* combination.

Three RAS genes, HRAS, NRAS, and KRAS, are the most frequently mutated oncogenes in cancer, with the most paramount mutation being the KRAS mutation (Hobbs et al., 2016). KRAS plays a certainly critical role in multiple signaling pathways for cell proliferation, differentiation, and survival, being acknowledged as an eminent tumor driver (Downward, 2003; van Hattum and Waldmann, 2014). KRAS mutations trigger the activation of KRAS by intervening in the normal RAS cycles between the GDP-bound inactive state and GTP-active state, shifting the equilibrium in favor of the GTP-active state (Hunter et al., 2015; Ostrem and Shokat, 2016). Among KRAS mutations, KRAS-G12C mutation is a single point mutation with a glycine-to-cysteine substitution at codon 12, which can be observed in colorectal and pancreatic cancers, and is particularly prevalent in non-small cell lung cancer (NSCLC) (Shin et al., 2019). The attempts toward recognizing small-molecule inhibitors for KRAS failed over years, consequently, KRAS was considered as an undruggable target (Downward, 2003; Wang et al., 2012; Papke and Der, 2017). Recently, covalent inhibitors targeting specific KRAS-G12C mutation have been developed and shown satisfactory preclinical efficacy in KRAS-G12C mutated tumor models (Patricelli et al., 2016; Fell et al., 2018). ARS-1620, as a potent and covalent specific inhibitor of KRAS-G12C, has an encouraging effect on tumor regressions in the KRAS-G12C mutated cancer cell line (Janes et al., 2018) and its outstanding anti-tumor activity in the mouse xenograft model offered the first *in vivo* verification of a potential approach that directly covalent targeting of KRAS-G12C mutation (Janes et al., 2018). As a preclinic drug, ARS-1620 has been also reported to disrupt the association between KRAS and Argonaute two on the plasma membrane, which may bring the therapeutic feasibility for pancreatic cancer (Shankar et al., 2020).

There has been considerable research regarding the relationship of expression levels of ABC transporters with KRAS mutations in cancer cells (Mohelnikova-Duchonova et al., 2013; Wei et al., 2016), while the link between the level of ABC transporters and KRAS inhibitors is poorly understood. In this study, we investigated the connection between novel emerged KRAS-G12C inhibitor, ARS-1620, and ABCB1-overexpressing cancer cells. Stirringly, we found that the overexpression of ABCB1 confers resistance to ARS-1620. Furthermore, our study provides the possible scenario in which the cancer cells might develop resistance to ARS-1620 *via* ABCB1 overexpression, and the possible solution for this scenario is the combination therapy of ARS-1620 with an ABCB1 inhibitor.

## Materials and Methods

### Chemicals

ARS-1620 was a free sample from ChemieTek (Indianapolis, IN). The monoclonal antibodies against ABCB1 (clone F4, Cat # SAB4200775) and other chemicals were purchased from Sigma Chemical Co (St. Louis, MO) except otherwise specified. Antibiotics (penicillin/streptomycin), trypsin-EDTA, and fetal bovine serum (FBS) were obtained from Hyclone (Waltham, MA, United States). The Horseradish peroxidase (HRP)-conjugated rabbit anti-mouse IgG secondary antibody (Cat # 7076S, lot #: 32) was the product of Cell Signaling Technology Inc. (Danvers, MA, United States). The GAPDH loading control monoclonal antibody (GA1R) (Cat # MA5-15738, lot #: SA247966), Alexa Fluor 488 conjugated goat anti-mouse IgG cross-adsorbed secondary antibody (Cat # A32723) were acquired from Thermo Fisher Scientific Inc. (Rockford, IL, United States). [^3^H]-paclitaxel (15 Ci/mmol) was obtained from Moravek Biochemicals, Inc. (Brea, CA).

### Cell Lines and Cell Culture

All the cells were cultured as previously described (Zhang et al., 2020). In brief, the human epidermoid carcinoma cell line KB-3-1 and its colchicine-induced ABCB1-overexpressing KB-C2 cell line, human colon carcinoma cell line SW620, and its doxorubicin-induced ABCB1-overexpressing SW620/Ad300 cell line were used (Lyall et al., 1987; Lai et al., 1991). The KB-3-1 and KB-C2 were not KRAS-G12C mutation cell lines, the SW620 and SW620/Ad300 were KRAS-G12V mutation but not KRAS-G12C mutation cell lines. HEK293/pcDNA3.1, HEK293/ABCB1, HEK293/ABCG2, and HEK293/ABCC1 were human embryonic kidney HEK293 cells transfected with empty vector pcDNA3.1, full-length *ABCB1*, full-length *ABCG2*, and full-length *ABCC1*, respectively (Fung et al., 2014). The moist incubator (37°C, 5% CO_2_) was used to culture the above cell lines.

### Cell Viability Assay

Cell survival percentage after treatment with ARS-1620 and other substrates was determined by MTT assay as described earlier (Dong et al., 2020). In short, the cells were seeded into 96-well plates (5×10^3^/well). After cell attachment, the cells were treated with serial diluted ARS-1620 or other chemotherapeutic agents in the presence or absence of ABCB1 inhibitor verapamil for 68 h. Then, MTT solution was added for another 4 h incubation. Later, the supernatant was discarded and DMSO was added to dissolve the formazan. The optical density was obtained by reading the plate at 570 nm with a microplate analyzer.

### Immunoblotting Assay

We performed Western blotting as described earlier (Feng et al., 2020). Briefly, cells were treated with ARS-1620 (3 μM) at 37°C for 0, 24, 48, or 72 h, and then lysed. Protein concentration in each group was determined by BCA protein quantitative method. An equivalent amount (20 μg) of protein was subjected to SDS-PAGE followed by transfer to the PVDF membranes. Next, blocking of the membranes were processed in 5% skimmed milk at room temperature for 2 h. Primary antibodies (ABCB1 or GAPDH) (1:1000) were applied to membranes at 4°C overnight. After washing the membranes with TBST 3 times, the HRP-conjugated anti-mouse antibody (1:1000) was incubated with the membranes at room temperature for 2 h. ECL chemiluminescence kit was used and the signal was captured by X-ray film. The analysis of protein expression levels was performed by ImageJ (NIH, MD).

### Immunofluorescence Assay

Immunofluorescence was carried out as previously described (Wang JQ. et al., 2020). Cells (1×10^5^) were incubated in 24-well plates with ARS-1620 (3 μM) for 0, 24, 48, or 72 h. The cells were fixed and permeated, then blocked with 6% BSA and incubated overnight with ABCB1 antibody (1:1000). Later, Alexa Fluor 488 IgG secondary antibody (1:1000) was applied to the cells for 2 h, and nuclei were re-stained with DAPI. The image was captured by EvoS FL fluorescence microscope (Life Technologies, MD).

### HPLC Accumulation Assay

KB-3-1 and KB-C2 cells (3×10^5^) were seeded into 6-well plates with 2 ml complete DMEM. After 2 days incubation, the medium was replaced with plain DMEM with or without 3 μM verapamil and incubated for 2 h, followed by adding 30 μM ARS-1620 to the wells for another 2 h. Afterwards, the cells were washed with PBS twice then lysed with 0.5% sodium dodecyl sulfate and acetonitrile. Samples were harvested and centrifuged at 14,000 rpm for 10 min. The supernatant was collected for intracellular concentration analysis using HPLC.

### [^3^H]-Paclitaxel Accumulation Assay

24-well plates were used for the culture of the cells (1×10^5^). On the second day, the cells were treated for 2 h with or without ARS-1620 or verapamil. Afterward, [^3^H] -paclitaxel was added to the specified wells and incubated for 2 h. After washing with PBS, the cells were gathered and shifted to scintillation vials and the radioactivity of different treatments were measured using Packard Tricarb 1900CA liquid scintillation analyzer (Packard Instrument, Dners Grove, IL).

### ATPase Assay

As previously described, the vanadate-sensitive ATPase activity of ABCB1 was measured in terms of the amount of inorganic phosphate (P_i_) generated by hydrolysis of ATP (Sarkadi et al., 1992). The quantity of P_i_ was determined using the improved colorimetric method by Murphy and Riley (Ojida et al., 2004).

### Molecular Modeling

Docking analysis was conducted with software Maestro 11.5 (Schrödinger, LLC, New York, NY, 2018) (Cai et al., 2020). Ligand preparation was essentially performed with the default protocol. Human ABCB1 (PDB ID: 6QEX) (Alam et al., 2019) protein preparation was conducted to optimize the structure, remove waters, and minimize the energy. Subsequently, a grid of 20 Å at the binding pocket of ABCB1 protein was generated. Glide XP docking was performed, followed by induced-fit docking (IFD) was carried out using the default protocol.

### Statistical Analysis

All data are expressed as mean ± SD and were acquired from a minimum of three independently repetitions. Statistical analysis was based on one-way ANOVA followed by the Dunnett’s test. *p*-value below 0.05 represents significant differences.

## Results

### The Cytotoxicity of ARS-1620 Was Decreased in ABCB1-Overexpressing Cells but Not in ABCG2- or ABCC1-Overexpressing Cells

**FIGURE 1:**
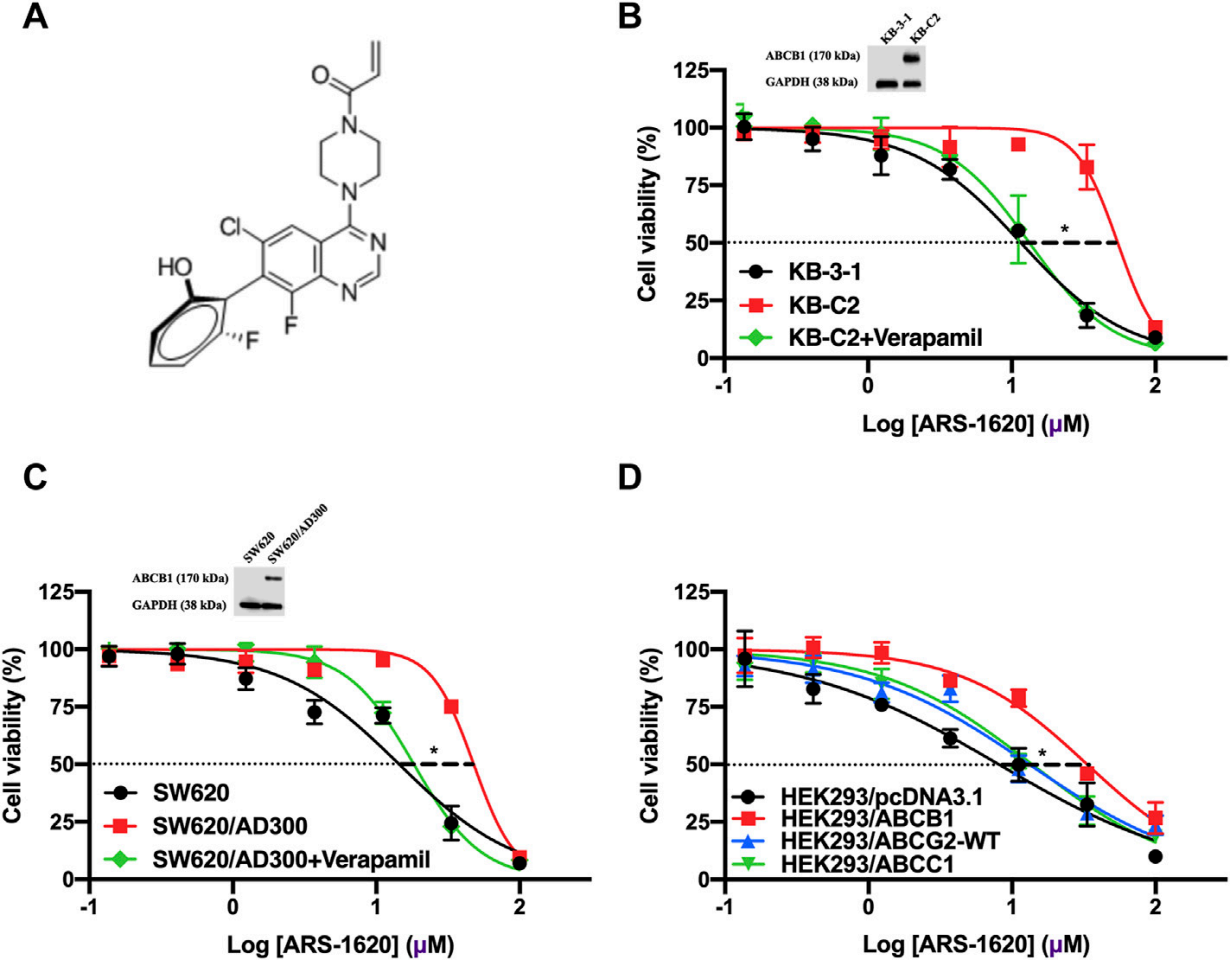
Cytotoxicity curves of ARS-1620 in parental and ABCB1-overexpressing cancer cells. **(A)** Chemical structure of ARS-1620. **(B, C)** KB-3-1, KB-C2, SW620, and SW620/Ad300 cells. The corresponding ABCB1 expression level were also displayed. **(D)** HEK293/pcDNA3.1, HEK293/ABCB1, HEK293/ABCG2 and HEK293/ABCC1 cells. Verapamil of 3 μM was used as positive control. Data are displayed as mean ± SD with three independent assays. *p < 0.05.

Firstly, we conducted the MTT assay to examine the cytotoxicity of ARS-1620 (the chemical structure is presented in Figure 1A) in KB-3-1, KB-C2, SW620, and SW620/Ad300 cell lines. As Figures 1B,C showed, the parental KB-3-1 and SW620 cells were significantly more sensitive to ARS-1620 than their drug-selected KB-C2 and SW620/Ad300 cells that overexpressing ABCB1. To further certify our observation, the cytotoxicity of ARS-1620 was also tested in gene-transfected cells HEK293/pcDNA3.1 and HEK293/ABCB1 (Figure 1D). Consistent with our previous results, HEK293/ABCB1 was more resistant and less sensitive to ARS-1620 compared with its parental cells HEK293/pcDNA3.1. Meanwhile, the cell survival curve of wild-type HEK293/ABCG2 and HEK293/ABCC1 cells displayed no significant increased sensitivity or reduced sensitivity. Additionally, the IC_50_ values (concentrations for 50% inhibition) of ARS-1620 and resistance fold (RF) were summarized in Table 1. The KB-C2 and SW620/Ad300 cells, compared with their parental KB-3-1 and SW620 cells, possessed 6.30- and 2.95-fold resistance to ARS-1620, respectively. HEK293/ABCB1 cells showed 3.07-fold resistance to ARS-1620 compared with HEK293/pcDNA3.1. These results indicated the possibility that the overexpression of ABCB1 decreased the cytotoxicity of ARS-1620, which renders the ability of ABCB1-overexpressing cells to survive with high concentrations of ARS-1620.

**TABLE 1:** The cytotoxicity of ARS-1620 on ABCB1-overexpressing cancer cells.

Cell lines	IC_50_ ± SD^a^ (RF^b^) (μM)					
	Doxorubicin	Doxorubicin + verapamil 3 μM	Paclitaxel	Paclitaxel + verapamil 3 μM	ARS-1620	ARS-1620 + verapamil 3 μM
KB-3-1	0.023 ± 0.010 (1.00)	0.030 ± 0.007 (1.31)	0.006 ± 0.001 (1.00)	0.005 ± 0.002 (0.83)	9.300 ± 3.605 (1.00)	11.001 ± 1.082 (1.18)
KB-C2	1.546 ± 0.047 (66.48)	0.021 ± 0.004 (0.91)*	2.961 ± 0.350 (534.10)	0.126 ± 0.020 (22.70)*	58.566 ± 2.363 (6.30)	25.020 ± 3.971 (2.69)^*^
SW620	0.090 ± 0.020 (1.00)	0.094 ± 0.006 (1.05)	0.051 ± 0.004 (1.00)	0.060 ± 0.013 (1.18)	14.916 ± 1.120 (1.00)	12.923 ± 1.737 (0.87)
SW620/Ad300	5.329 ± 0.092 (59.18)	0.795 ± 0.139 (8.83)*	3.522 ± 0.122 (69.01)	0.342 ± 0.106 (6.69)*	44.063 ± 3.695 (2.95)	14.751 ± 1.652 (0.99)*
HEK293/pcDNA3.1	0.078 ± 0.011 (1.00)	0.107 ± 0.044 (1.37)	0.045 ± 0.008 (1.00)	0.059 ± 0.009 (1.37)	10.991 ± 1.700 (1.00)	11.380 ± 2.485 (1.04)
HEK293/ABCB1	2.382 ± 0.703 (30.58)	0.062 ± 0.009 (0.80)*	1.610 ± 0.222 (35.65)	0.057 ± 0.019 (1.26)^*^	33.739 ± 7.157 (3.07)	12.027 ± 3.322 (1.09)*

a IC_50_ values were represented as mean ± SD acquired from at least three independent experiments.

b Resistance fold (RF) was calculated from dividing the IC_50_ values of parental or resistant cells in each treatment by the IC_50_ values of corresponding parental cells with doxorubicin, paclitaxel or ARS-1620, and without verapamil.

*p < 0.05 versus control treatment.

### The Reinstated Sensitivity of ABCB1-Overexpressing Cells to ARS-1620 by the Introduction of Verapamil

Towards to get more supporting evidence that the overexpression of ABCB1 transporter leads to ARS-1620 resistance in ABCB1-overexpressing cells, verapamil, the pervasive used first-generation inhibitor of ABCB1, was used to co-treat with ARS-1620 in KB-C2, SW620/Ad300, and HEK293/ABCB1 cells. As shown in Figures 1B,C and Table 1, in the presence of 3 μM verapamil, the resistance to ARS-1620 was significantly reversed in KB-C2, SW620/Ad300, and HEK293/ABCB1 cells, the resistance fold dropped to 2.69-, 0.99-, and 1.09-fold, respectively. These results reinforced our assumption that the overexpression of ABCB1 exists as one predominant contributor to the resistance of ARS-1620.

### Higher Concentrations of ARS-1620 Boosted the Accumulation of [^3^H]-Paclitaxel

**FIGURE 2:**
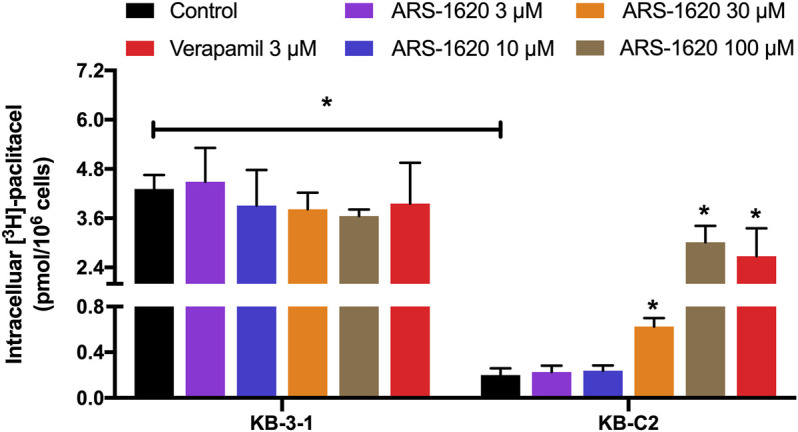
ARS-1620 affected Intracellular [^3^H]-paclitaxel accumulation in ABCB1-overexpressing cancer cells. Different concentrations (3, 10, 30, 100 μM) of ARS-1620 were used in ABCB1-overexpressing cell lines. Results are expressed as mean ± SD of three independent assays. * represents *p* < 0.05.

Next, we tested the accumulation of intracellular [^3^H]-paclitaxel when the cells were co-treated with distinct concentrations of ARS-1620 (3, 10, 30, 100 μM) over a short period. As observed in Figure 2, ARS-1620 at the concentration of 3 μM or even the toxic concentration of 10 μM did not influence the accumulation of [^3^H]-paclitaxel in KB-C2 cells. However, when the concentrations of ARS-1620 reached 30 and 100 μM, the [^3^H]-paclitaxel level was significantly increased in KB-C2 cells. In addition, the accumulation of [^3^H]-paclitaxel in parental KB-3-1 cells was not perturbed by ARS-1620. Notably, the toxic concentrations chosen in this assay were unlikely to affect the viability and function of the parental and resistant cells in this short treatment period. The restored accumulation of [^3^H]-paclitaxel in resistant cell treating with high concentrations of ARS-1620 implied that high concentrations of ARS-1620 might compete with paclitaxel for binding to substrate binding site, providing potential and indirect evidence that ARS-1620 is a substrate of ABCB1.

### ARS-1620 Dramatically Stimulated ATPase Activity of ABCB1

**FIGURE 3:**
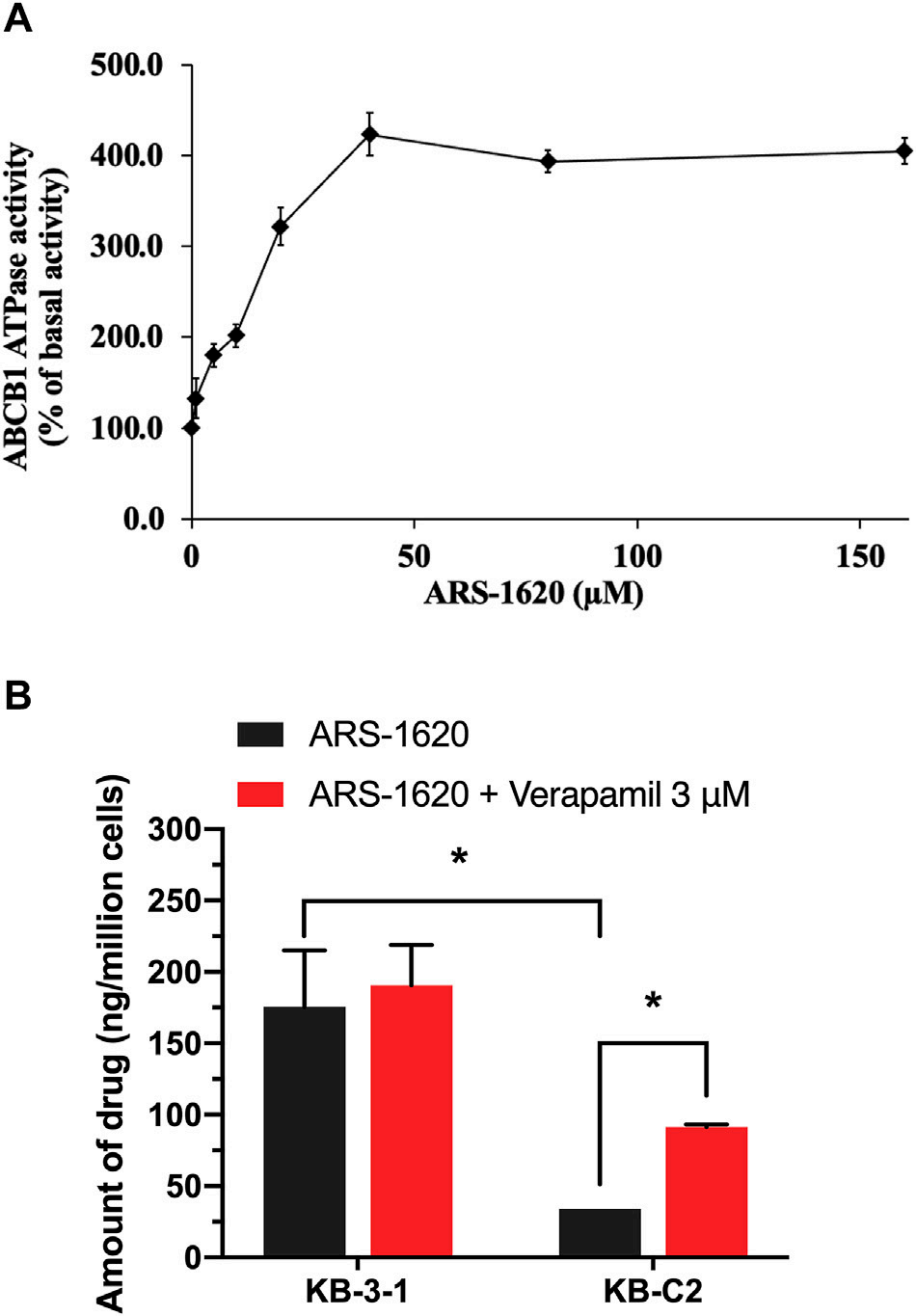
The effect of ARS-1620 on the ATPase activity of ABCB1 and ARS-1620 got actively transported out by ABCB1-overexpressing cancer cells. **(A)** ARS-1620 (0–160 μM) stimulates ABCB1 ATPase activity. **(B)** The intracellular accumulation of ARS-1620 in KB-3-1 and KB-C2 cells. Data are displayed as mean ± SD of three independent assays. *p < 0.05.

The ATPase activity was considered a non-negligible factor affecting the function of ABCB1, since the energy supplied to pump out the substrate drugs was generated through the hydrolysis of ATP. Thus, we measured the effect of ARS-1620 (0–160 μM) on the ATP hydrolysis in ABCB1 membrane vesicles. As displayed in Figure 3A, ARS-1620 showed a stimulatory effect on ABCB1-associated ATPase activity in a dose-depend manner. The stimulatory effect of ARS-1620 reached 50% maximal (EC_50_) at 14.8 μM. Besides, a maximum of 4.23-fold of the basal activity was achieved by ARS-1620. Understanding that stimulation of ATPase activity is commonly relevant to ABC-mediated substrates transport, the above results illustrated that ARS-1620 may act as a substrate of ABCB1 transporter which was in accord with the cytotoxicity results.

### The Decreased Intracellular Accumulation of ARS-1620 in ABCB1-Overexpressing Cells

For further verification of ARS-1620 is a substrate of ABCB1 transporter, an HPLC assay was performed to examine the concentration of ARS-1620 with or without adding verapamil in KB-3-1 and KB-C2 cells. In Figure 3B, the amount of ARS-1620 was 5-times higher in KB-3-1 than KB-C2. After co-incubation with verapamil, the concentration of ARS-1620 was significantly increased in KB-C2 cells compared with KB-3-1 cells. These results provided direct and strong evidence that ARS-1620 resistance was conferred by overexpression of ABCB1 and efflux by ABCB1.

### ARS-1620 Could not Reverse ABCB1-Mediated MDR

**FIGURE 4:**
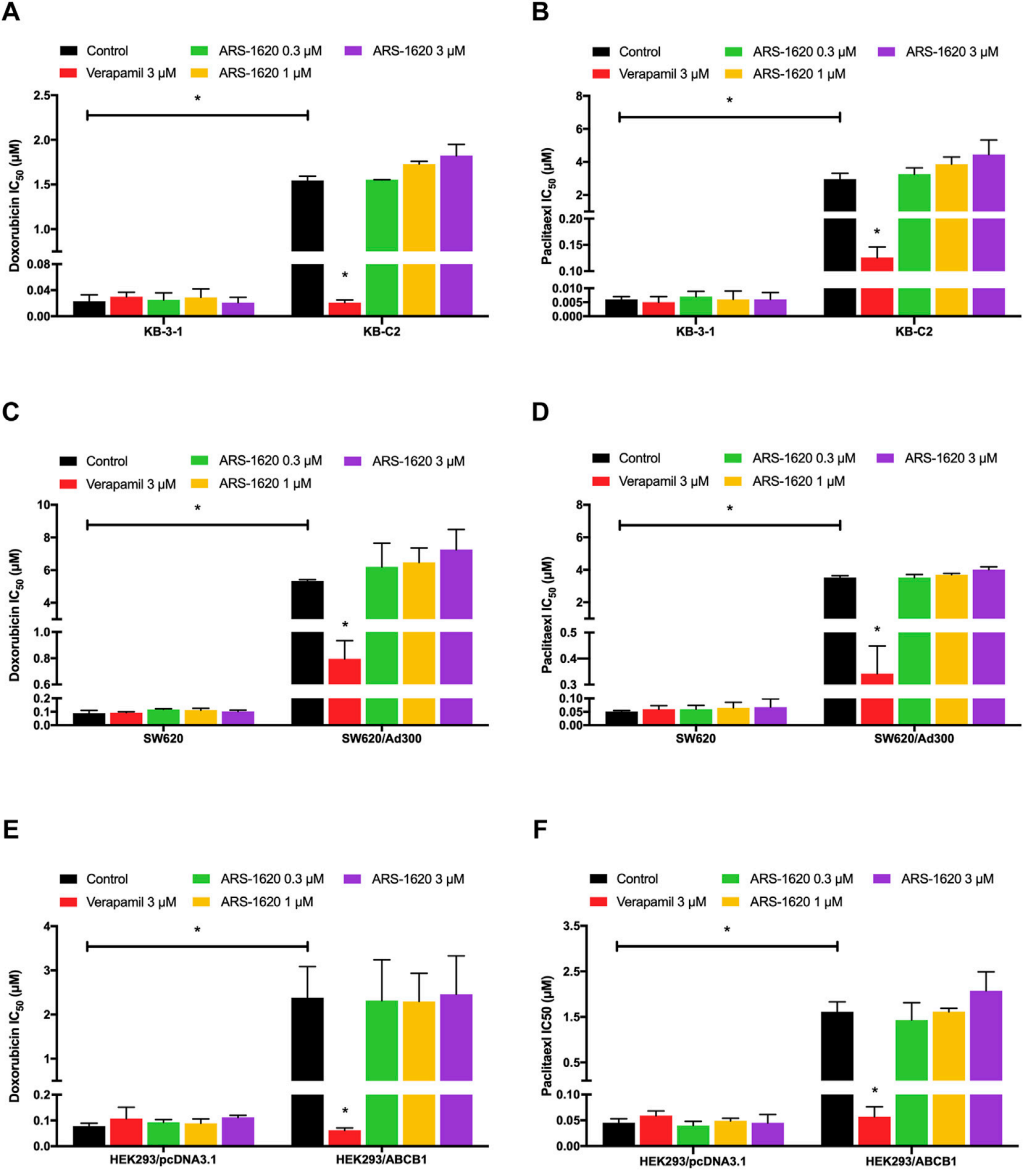
Non-toxic concentrations of ARS-1620 were not able to reverse ABCB1-mediated MDR. In parental **(A, C, E)** and resistant **(B, D, F)** cell lines, ARS-1620 (0.3, 1, 3 μM) did not re-sensitize ABCB1-overexpressing cells to ABCB1 substrates (doxorubicin and paclitaxel) like verapamil, the positive control. *p < 0.05.

It has been reported that some substrates could act as reversal reagents that competed with other conventional substrates like paclitaxel or doxorubicin at the substrate-binding site of ABCB1 transporter (Wang J. et al., 2020; Wu et al., 2020), resulting in a higher concentration of paclitaxel or doxorubicin remained in cells. Therefore, reversal experiments were carried out in three pairs of cell lines to examine this potentiality. Based on the results of the cytotoxicity assay (Figure 1), the non-toxic concentration of ARS-1620 (0.3, 1, 3 μM) was selected. As shown in Figure 4, the IC_50_ values of doxorubicin and paclitaxel were slightly but not significantly increased in drug resistant cells by the addition of ARS-1620 in a concentration-dependent manner which signifies that ARS-1620 could not behave as an inhibitor of ABCB1 at non-toxic concentrations. The verapamil that significantly re-sensitized the resistant cells was used as a positive inhibitor of ABCB1.

### ARS-1620 has no Significant Effect on the Expression or Localization of ABCB1 Protein

**FIGURE 5:**
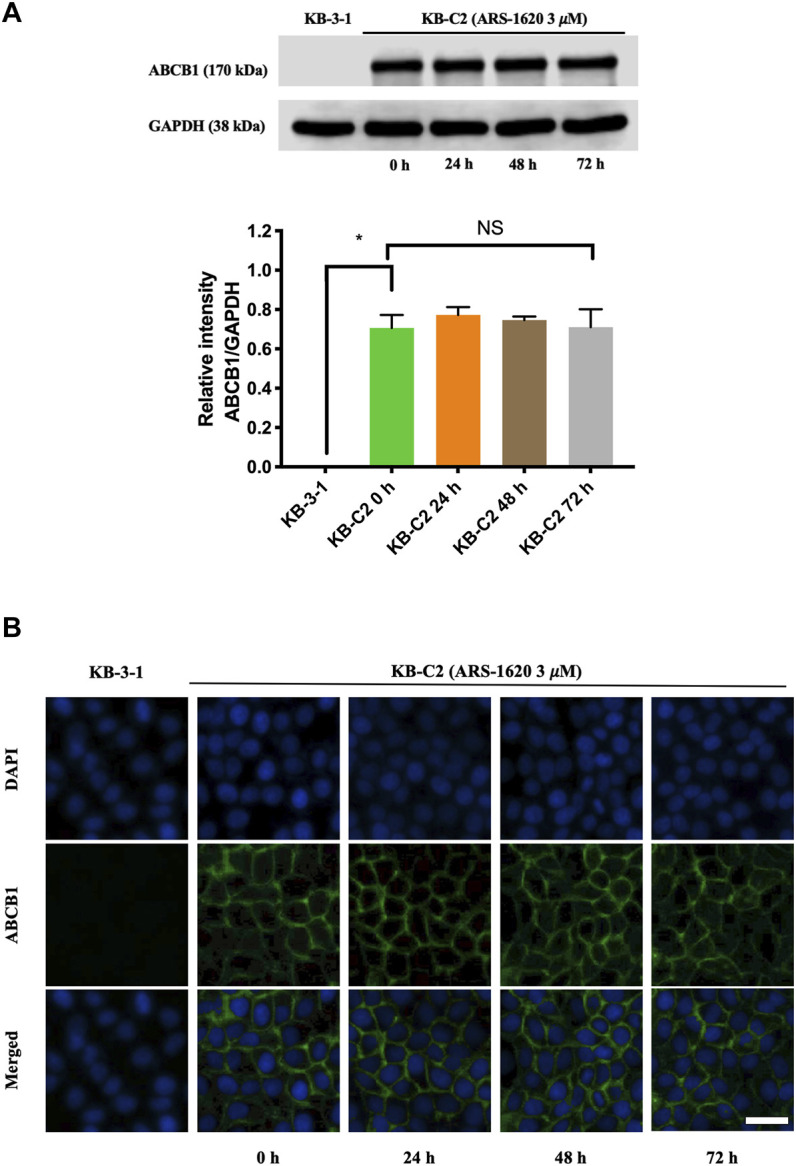
ARS-1620 did not influence the protein expression level and localization of ABCB1. **(A)** The ABCB1 expression level in KB-C2 cells with ARS-1620 (3 μM) for 0, 24, 48, and 72 h. **(B)** The captured images displayed subcellular localization of ABCB1 transporter with ARS-1620 (3 μM) for 0, 24, 48 and 72 h. Green showed ABCB1; blue showed DAPI counterstains the nuclei; Scale bar: 200 μm. Results are showed as mean ± SD of three independent experiments. NS indicates no significance.

Knowing that substrate drugs may interact with ABCB1 protein which brings about increasing the protein expression of ABCB1 or altering the localization of ABCB1 on the cell membrane (Wu et al., 2020), we conducted immunoblotting and immunofluorescence to investigate these potentialities. According to Figure 5A, the non-toxic concentration of ARS-1620 (3 μM) did not affect the expression level of ABCB1 in KB-C2 cells after comparing the relative intensity of groups with different treating periods (0, 24, 48, 72 h). Furthermore, the results from Figure 5B showed that ABCB1 protein still located on the cell membrane without alterations during the 24–72 h treating periods with ARS-1620. The parental KB-3-1 cells in these experiments were set as the negative control.

### ARS-1620 Possessed a High-Affinity Score With the Human ABCB1 Model in the Docking Study

**FIGURE 6:**
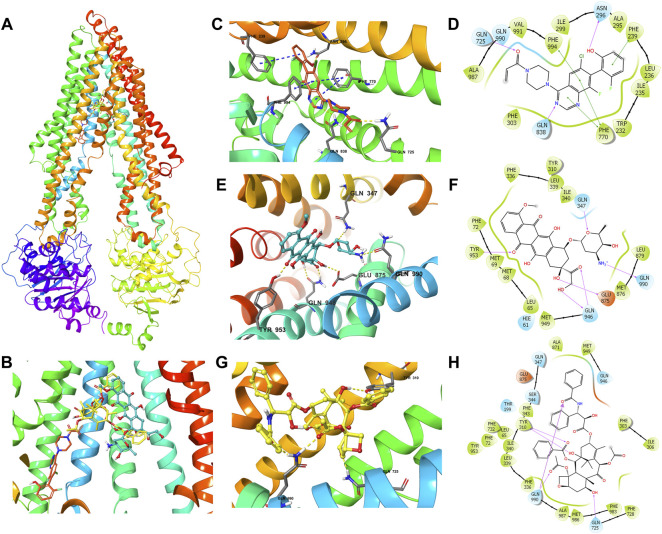
The molecular modeling of ARS-1620, doxorubicin, and paclitaxel with human ABCB1. **(A)** The binding site of ARS-1620 (orange), doxorubicin (cyan), and paclitaxel (yellow) within human ABCB1. **(B)** The enlarged diagram of ARS-1620, paclitaxel, and doxorubicin within the binding site of human ABCB1. The interactions of ARS-1620 **(C)**, doxorubicin **(E)**, and paclitaxel **(G)** with the ABCB1 model in the three-dimension diagram. Ligands are shown as ball and stick mode: nitrogen—blue, oxygen—red, hydrogen—white, chloride—green, fluoride—light cyan. Interactions are indicated: hydrogen bonds—yellow dotted short line, π-π stacking—blue dotted short line. The interactions of ARS-1620 **(D)**, doxorubicin **(F)**, and paclitaxel **(H)** with the ABCB1 model in the two-dimension diagram: purple arrow hydrogen bond, green short line—π-π interaction.

The results showed that ARS-1620, doxorubicin, and paclitaxel have high binding affinities with human ABCB1 (Figure 6) with docking scores -12.807, -12.241, and -15.527 kcal/mol, respectively. As exhibited in Figures 6C,D, the interactions between ARS-1620 and ABCB1 include hydrogen bonds and π-π interaction. The three aromatic rings of ARS-1620 are involved in the π-π interactions with the residues Phe239, Phe770, and Phe994. The carbonyl group and a nitrogen atom on quinazoline ring ARS-1620 formed hydrogen bonds with Gln725 and Gln838 as hydrogen-bond acceptors, while the hydroxyl group interacted with Asn296 as hydrogen bond donor. Distinct from ARS-1620, paclitaxel and doxorubicin with more oxygen atoms in their structures, tended to form hydrogen bonds rather than π-π interactions with ABCB1. Residues Gln347, Glu 875, Gln946, Tyr953, and Gln990 formed hydrogen bonds with doxorubicin. Gln946 interacted with a carbonyl group and a hydroxyl group as hydrogen bond donor and acceptor, respectively. Interestingly, the amino group became a cation and interacted with Gln990 with a hydrogen bond. Due to the acidic cancer microenvironment (Boedtkjer and Pedersen, 2020), the amino group can become protonated to a cation, and bind with the residues. Paclitaxel formed hydrogen bonds with residues Tyr310, Gln725, and Gln990, where Tyr310 interacted with both carbonyl and hydroxyl groups.

## Discussion

RAS is the most common oncogene in cancer, and KRAS mutations are the predominant oncogene among the three RAS subtypes (HRAS, NRAS, and KRAS) (Hobbs et al., 2016), which was regarded as “untreatable” targets for a long time. To devise treatment strategies, the downstream effector pathways have been extensively studied, for instance, the ERK MAPK cascade (Ryan and Corcoran, 2018; Ryan et al., 2020). However, recently, covalent inhibitors targeting specific KRAS mutations - glycine 12 to cysteine (G12C) have been developed, providing an unprecedented opportunity to directly target KRAS and showing encouraging preclinical efficacy in KRAS-G12C tumor models (Patricelli et al., 2016; Fell et al., 2018; Janes et al., 2018). Two KRAS-G12C inhibitors have completed preliminary safety assessments in phase 1 clinical trials: AMG510 (NCT03600883) and MRTX1257 (NCT03785249). As the first two drugs to directly inhibit mutated KRAS, they offer an unprecedented therapeutic opportunity to target this key oncogene. ARS-1620, another newly developed mutant-specific inhibitor of KRAS-G12C, has a strong inhibitory effect on KRAS-G12C mutant tumors (Molina-Arcas et al., 2019). At the same time, the overexpression of the ABC transporter is one of the causes of MDR in tumor cells leading to chemotherapy failure, and while the relationship between ABC expression level and KRAS mutation in cancer cells can be confirmed (Mohelnikova-Duchonova et al., 2013; Wei et al., 2016), the relationship between the expression level of ABC transporters and KRAS inhibitor is not clear, and the effect of ARS-1620 on ABCB1-mediated MDR has not been studied yet. Therefore, we investigated the potential interaction of ABCB1 with ARS-1620.

In this study, we surveyed the connection of ARS-1620 with ABCB1-overexpressing cancer cells and we found that the overexpression of ABCB1 confers resistance to ARS-1620, which may affect its effectiveness in clinical anticancer treatments. Cell viability was measured in both drug selected ABCB1-overexpressing cells and *ABCB1* gene transfected cells. The ABCB1-overexpressing KB-C2 and SW620/Ad300 were highly resistant to doxorubicin, paclitaxel, as well as ARS-1620. Considering drug-induced MDR has a variety of mechanisms, we also used ABCB1-transfected HEK293/ABCB1 cells which the overexpression of ABCB1 was a unique element resulting in MDR. Compared with the parental cells, the ABCB1-overexpressing cell lines were more resistant to ARS-1620, and the results suggested that the overexpression of ABCB1 could reduce the cytotoxicity of ARS-1620, which may eventually lead to its drug resistance. Furthermore, co-treatment with the ABCB1 inhibitor verapamil restored the sensitivity of ABCB1-overexpressing cells to ARS-1620, a result similar to other substrate drugs, such as ceritinib (Katayama et al., 2016). Additionally, the results of MTT assay in HEK293/ABCG2 and HEK293/ABCC1 revealed that ARS-1620 was not the substrate of these two transporters.

To better validate it, we further tested the accumulation of intracellular [^3^H]-paclitaxel when the cells were co-treated with ARS-1620 (3, 10, 30, 100 μM) over a short period. Results showed that ARS-1620 at the concentration of 3 μM or even the toxic concentration of 10 μM did not influence the accumulation of [^3^H]-paclitaxel in KB-C2 cells. However, when the concentrations of ARS-1620 reached 30 and 100 μM, the [^3^H]-paclitaxel level was significantly increased in KB-C2 cells. Moreover, the accumulation of [^3^H]-paclitaxel in parental KB-3-1 cells was not perturbed by ARS-1620, suggesting that ABCB1 was the main cause for the decrease of substrate accumulation in drug-resistant cells. Since ABC transporters utilize the energy generated by ATP catabolism to pump out exogenous substrate drugs, leading to decreased concentration of effective substrate drugs in cells, weakened efficacy, and the development of drug resistance (Bloise et al., 2016), the effect of ARS-1620 on ATPase activity of ABCB1 was measured by ATPase assay. The results showed that ARS-1620, like other substrates, significantly stimulated the activity of ABCB1-related ATPase in a concentration-dependent manner. Taken the previous results together, we concluded ARS-1620 is a substrate of the ABCB1 transporter. Later, the results of the HPLC assay confirmed our assumption that ARS-1620 was positively transported by KB-C2 cells compared with KB-3-1 cells and this outcome could be reversed by verapamil. This measurement of intracellular ARS-1620 accumulation directly offers compelling evidence that ARS-1620 is transported by ABCB1 as a substrate. After determining ARS-1620 is a substrate of ABCB1, it drew our attention that whether it can act as an inhibitor to reverse ABCB1 overexpression-associated MDR, as it has been investigated that certain substrates possess such ability (Levy et al., 2019). MTT assay was conducted with non-toxic ARS-1620, while no significant difference was found between various treatments. These results implied that high concentrations of ARS-1620 may serve as a competitive inhibitor to compete with paclitaxel for binding to substrate binding site, which also provided the indirect evidence that ARS-1620 is a substrate of ABCB1. In addition, usually substrate-drugs might affect the expression and location of corresponding ABC transporters to some extend (Yang et al., 2021). In our study, the ABCB1 expression level was not altered during 24–72 h of ARS-1620 treatment. This is probably because KB-C2 cells were already resistant to ARS-1620, rendering the induction effect of ARS-1620 ignorable. Then the immunofluorescence results showed that ARS-1620 did not affect the localization of ABCB1. However, we cannot rule out the possibility that longer incubation time may change the expression and cellular localization of the ABCB1 transporter protein. More in-depth studies such as whether ARS-1620 can induce ABCB1 expression in parental cells also should be further investigated.

Molecular docking is a theoretical simulation method that focuses on studying intermolecular interactions and predicting binding modes and affinities (Rosano et al., 2013; Lionta et al., 2014), aiming to find the optimal binding sites for substrate and receptor molecules and evaluate the binding strength between docked molecules. IFD analysis simulated the molecular interaction between ARS-1620 and the human ABCB1 drug-binding pocket and results suggested that ARS-1620 has a high binding affinity to ABCB1 with a docking score of −12.807 kcal/mol similar to the known ABCB1 substrates doxorubicin (-12.241 kcal/mol) and paclitaxel (−15.527 kcal/mol). Besides, the higher docking score of paclitaxel than ARS-1620 may indicate that paclitaxel is more potent to bind ABCB1 to be preferentially pumped out than ARS-1620, but ARS-1620 takes priority when the concentration of paclitaxel unchanged and ARS-1620 increased to a specific level. This could be the explanation of the results in our [^3^H]-paclitaxel accumulation assay. However, more investigations regarding the relationship of docking score and reality interaction should be done as molecular docking cannot serve as an exact predictor.

In conclusion, our study strongly demonstrates that the overexpression of ABCB1 is associated with the resistance of ARS-1620 in cancer cells, which provides a valuable basis for future using of ARS-1620 in clinical studies. It also should be noted that KB-3-1 and SW620 cells are not KRAS-12C mutant cells, and more in-depth studies of KRAS-12C mutant ABCB1-overexpressing cells will allow us to further our comprehensive understanding and establish advanced clinical strategies.

## Data Availability

The original contributions presented in the study are included in the article/supplementary material, further inquiries can be directed to the corresponding authors.
